# The Impact of Sunlight Exposure on Postoperative Hypoparathyroidism: A Retrospective Analysis from Two Greek Centers

**DOI:** 10.3390/jcm14134418

**Published:** 2025-06-21

**Authors:** Angeliki Chorti, Ioannis Pliakos, Moysis Moysidis, Aikaterini Smprini, Sohail Bakkar, Theodossis Papavramidis

**Affiliations:** 1 Propaedeutic Department of Surgery, AHEPA University Hospital, Aristotle University of Thessaloniki, 54636 Thessaloniki, Greece; tpapavra@auth.gr; 2 Department of Minimal Invasive Endocrine Surgery, Euromedica Kyanos Stavros, 54454 Thessaloniki, Greece; plliakos@hotmail.com (I.P.); moisisdoc@gmail.com (M.M.); ksmprini@hotmail.com (A.S.); 3 Endocrine & General Surgery, The Hashemite University, Amman 13133, Jordan; sohail.bakkar@gmail.com

**Keywords:** sunshine, postoperative hypoparathyroidism, postoperative hypocalcemia, vitamin D

## Abstract

**Background:** Postoperative hypoparathyroidism is a common complication of thyroid surgery. Sunlight is a natural source of ultraviolet B (UVB) radiation, which facilitates the synthesis of vitamin D3 in the skin. Inadequate sunlight exposure has been linked to vitamin D deficiency, potentially exacerbating the risk of hypocalcemia in patients undergoing thyroid surgery. The aim of the present study is to evaluate the effect of sunshine levels on postoperative hypoparathyroidism. **Method:** We retrospectively evaluated patients that underwent total thyroidectomies at two different centers (Thessaloniki and Rhodes) by the same surgical team from 2021 to 2023 in terms of postoperative hypoparathyroidism. We compared the sunshine levels at each center the year before surgery and correlated them with postoperative levels of parathyroid hormone, serum ionized calcium, and phosphorus. **Results:** One-hundred twenty patients (Group Thessaloniki = 60 patients, Group Rhodes = 60 patients) who were matched for demographic characteristics and type of thyroid disease and surgery were enrolled in our study. The sunshine levels were different between the two centers (Rhodes > Thessaloniki, *p* < 0.001). It was found that sunshine levels affect preoperative serum ionized calcium (*p* = 0.002) and postoperative parathyroid hormone levels (*p* = 0.025). **Conclusions:** Sunlight exposure levels may play a crucial role in preventing postoperative hypoparathyroidism. Patients living in locations with higher sunshine levels may have lower rates of postoperative hypoparathyroidism.

## 1. Introduction

Postoperative hypoparathyroidism is the most common complication after thyroid operations. It is divided into temporary hypoparathyroidism, with a reported incidence of 19–38%, and permanent hypoparathyroidism, with an incidence of 0–3% [1,2]. The risk factors for hypoparathyroidism development after thyroid surgery are multiple and distinct, including perioperative biochemical factors, surgery-related factors, patient-related factors, and disease-related factors [1,3]. Among them, vitamin D deficiency has been reported as an additional risk factor for postoperative hypoparathyroidism as well as a predictor for the development of hypocalcemia [1,2].

Vitamin D can either be produced in the human body or supplemented by nutrition [4]. Regarding self-production, the human body cannot independently produce vitamin D. Vitamin D synthesis starts in the human skin, which contains provitamin D3 7-dehydrocholesterol, a precursor of vitamin D. This precursor molecule is transformed into previtamin D3 by the penetration of ultraviolet B radiation (UVB) after sun exposure, which breaks the B ring and thus forms pre-D_3_ [5]. Parathyroid hormone, FGF23, calcium, and phosphate are the major regulators of renal 1-hydroxylase, the enzyme that takes part in 1,25(OH)2D production. Therefore, frequent sun exposure is crucial for vitamin D production and, thus, calcium metabolism.

The aim of the present study is to investigate the potential correlation between patient sunlight exposure and the incidence of postoperative hypoparathyroidism after thyroid operations.

## 2. Materials and Methods

### 2.1. Study Design

This is a retrospective cohort study. Patients that underwent thyroid surgery from July 2021 to December 2023 at two distinct medical centers (Euromedica Kyanos Stavros, Thessaloniki, Northern Greece, and Euromedica Geniki Kliniki, Rhodes, Southern Greece) were enrolled. The operations were performed by the same surgical team, composed of surgeons with expertise in endocrine surgery. Patients’ demographic data, types of operation, prior diagnosis, preoperative values of serum ionized calcium (Ca), phosphorus (P) levels, and parathyroid hormone (PTH) levels and postoperative values of serum ionized calcium, phosphorus, and parathyroid hormone at the 1st postoperative day were retrieved. Inclusion criteria were age > 18 years old and having undergone thyroid surgery (total thyroidectomy, total thyroidectomy, and central compartment lymph node dissection, and total thyroidectomy and central- and lateral-compartment lymph node dissection), while patients <18 years old, individuals who had undergone parathyroid surgery, and those with missing data were excluded. Data collection and analysis were performed according to the ethical standards of the 1975 Helsinki declaration and its later amendments and comparable ethical standards. Patients’ informed consent was waived because of the retrospective nature of this study, and ethical approval was obtained from the Local Ethical Committee (224/87/21.03.2024).

Patients were separated into two groups: (a) Group T, including patients that lived in Northern Greece and were operated on at the medical center of Thessaloniki, and (b) Group R, including patients that lived in Southern Greece and were operated on at the medical center of Rhodes. Patients in each group were matched based on age, gender, diagnosis, type of operation, preoperative 25(OH) vitamin D levels, preoperative vitamin D supplementation, and month of the year that the operation took place. Preoperative oral vitamin D supplementation protocol in Greece includes the administration of 25,000 IU of vitamin D for 3 weeks preoperatively. Hypoparathyroidism was defined as PTH < 15 pg/mL, and hypocalcemia was defined as serum albumin-corrected calcium levels < 8.5 mg/dL on the 1st postoperative day.

Data about sunshine levels in Thessaloniki and Rhodes were retrieved by the Greek National Meteorology Center. Mean sunshine levels for each month were registered by calculating the mean sunshine levels for the last 12 months before surgery.

### 2.2. Statistical Analysis

Quantitative variables that follow a normal distribution were reported as means ± standard deviations or medians (interquartile range) for those that did not follow a normal distribution. Qualitative variables were reported as frequencies and relative frequencies. Sample normality was tested using the Kolmogorov–Smirnov test. Not all of the continuous variables followed a normal distribution, so non-parametrical tests were used. To compare the median values of the quantitative variables in the categories of binary categorical variables, the Mann–Whitney test was used. Furthermore, using the Spearman correlation index (for non-normal distributions), possible correlations and dependencies between the variables were investigated. Statistical processing was performed using the statistical package SPSS version 26 (IBM), (SPSS Inc., Chicago, IL, USA). A *p*-value < 0.05 was set as the level of statistical significance.

## 3. Results

In total, 120 patients were enrolled in this study, with 60 in each group. The mean age was 47.13 ± 13.01 years old. Overall, 29.2% of the patients (35 patients) were male, and 70.8% (85 patients) were female, with a male-to-female ratio of 1:2.4. The indication for surgery was benign disease for 51.7% (62 patients) and malignant disease for 48.3% (58 patients). The type of operation was total thyroidectomy (TT) for 65% (78 patients), total thyroidectomy with central compartment lymph node dissection (TT + CC) for 26.7% (32 patients), and total thyroidectomy with central and lateral compartment lymph node dissection (TT + CC + LC) for 8.3% (10 patients). These parameters exhibited no statistically significant differences between groups, as shown in Table 1.

**Figure 1:**
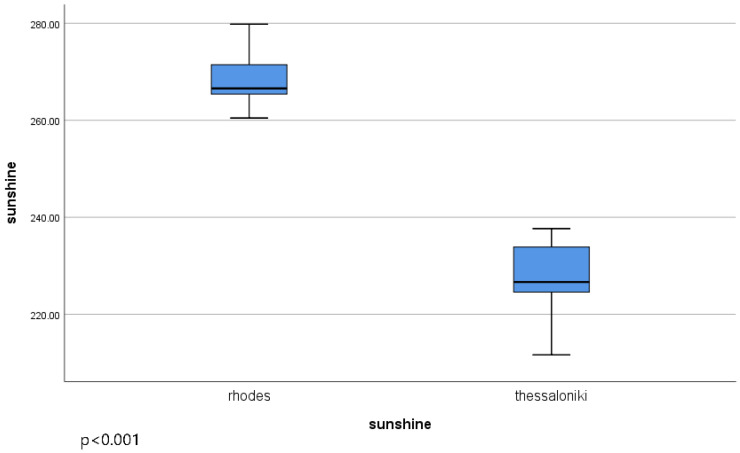
Sunshine levels in two different locations, Rhodes and Thessaloniki, with statistically significant differences (*p* < 0.001).

Median sunshine levels in Thessaloniki and Rhodes were compared. The median sunshine level in Thessaloniki was 226.6 h (IQR:9.36), while that in Rhodes was 266.5 h (IQR: 6.12). Comparing the sunshine levels between Thessaloniki and Rhodes revealed a statistically significant difference (t = 36.35, *p* < 0.001), as shown in Figure 1.

**Figure 2:**
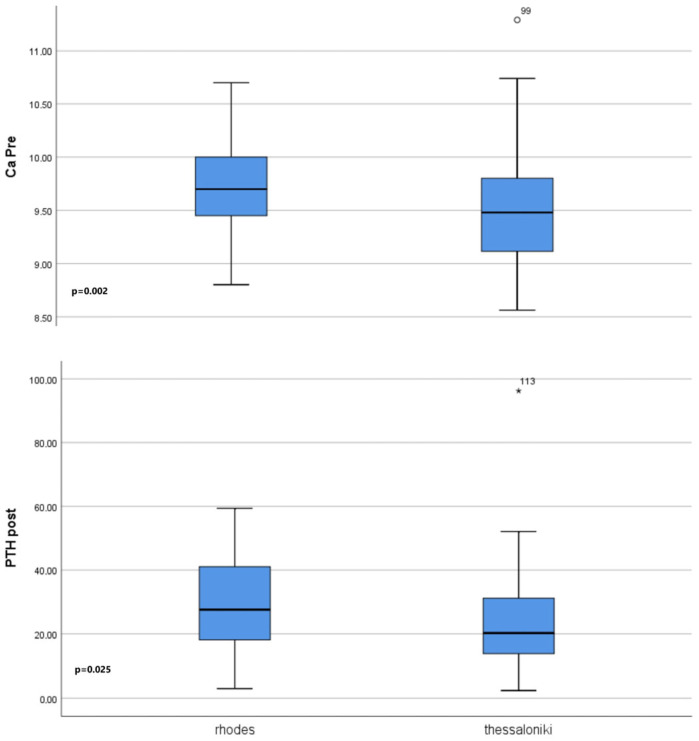
Preoperative levels of serum ionized calcium and postoperative levels of parathyroid hormone of patients from centers in Rhodes and Thessaloniki, with a statistically significant difference (*p* = 0.002, *p* = 0.025, respectively).

The sunshine levels in each group were compared to pre- and postoperative values of serum ionized calcium, phosphorus, and parathyroid hormone. There was a statistically significant difference between the groups for preoperative ionized calcium (*p* = 0.002), preoperative and postoperative phosphorus (*p* = 0.02 and *p* = 0.005), and postoperative parathyroid hormone levels (*p* = 0.025). The patients in Group R had higher preoperative serum ionized calcium and postoperative parathyroid hormone levels than those in Group T (Figure 2).

**Figure 3:**
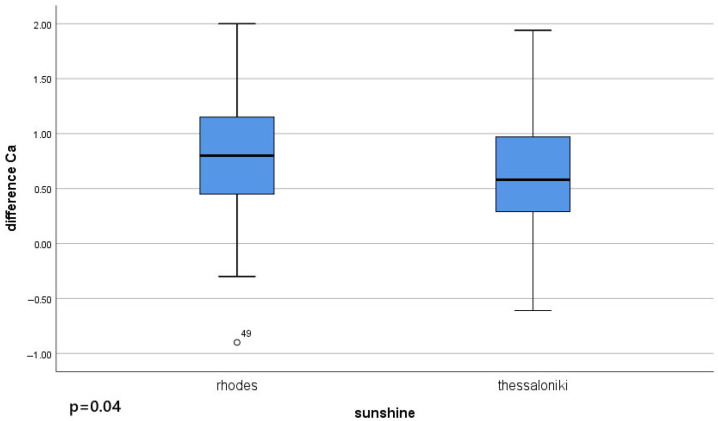
Correlation between the difference between pre- and postoperative serum ionized calcium levels in Rhodes and Thessaloniki and sunshine levels, with a statistically significant difference (*p* = 0.04).

Furthermore, the difference between pre- and postoperative values of serum ionized calcium and parathyroid hormone was compared to sunshine levels in each group. A statistically significant difference was found in the variation of serum ionized calcium pre- and postoperatively correlated with sunshine levels (*p* = 0.04) (Figure 3).

## 4. Discussion

Our study reveals the influence of different sunshine levels at different locations on perioperative biochemical factors related to postoperative hypoparathyroidism development. Higher sunshine levels correlate with higher preoperative serum ionized calcium and postoperative parathyroid hormone levels, leading to a lower incidence of postoperative hypoparathyroidism after thyroid surgery.

The role of ultraviolet B radiation (UVB) in vitamin D synthesis is well established [5]. UVB exposure and thus sunshine exposure differ among European countries, as confirmed by O’ Neill et al. [6]. Season, latitude, and weather conditions can influence UVB exposure and thus vitamin D levels. Vitamin D produced in the skin can provide 80–100% of the human body’s requirements. The higher the latitude, the lower the UVB availability. So, Northern European countries seem to have a greater need for oral vitamin D supplementation than southern countries [6,7]. In our study, we found that even in the same country, patients from northern parts can have less UVB exposure than those from southern parts.

In the international literature, there are no studies that directly examine the correlation between sunshine levels and postoperative hypoparathyroidism development. Moreover, studies about vitamin D deficiency and the prediction of postoperative hypoparathyroidism have yielded inconclusive results. The American Thyroid Association (ATA) guidelines have introduced preoperative vitamin D deficiency as a risk factor for postoperative hypoparathyroidism, as discussed in other published studies [1,8]. Vitamin D is crucial for proper calcium absorption following an operation [1]. Two meta-analyses from 2021 concluded that decreased preoperative levels of 25-hydroxy vitamin D can be a poor predictor of postoperative hypoparathyroidism [9,10]. A study by Vaitsi et al. has also pointed out that different levels of vitamin D deficiency (mild, moderate, and severe) have different effects on postsurgical hypoparathyroidism, and thus only severe vitamin D deficiency results in permanent hypoparathyroidism [9]. Severe deficiency was correlated with a higher percentage of hypoparathyroidism than states with sufficient levels of vitamin D [11]. Vitamin D deficiency was not found to be a risk factor for hypoparathyroidism in a Greek study, but it should be mentioned that the patients in both study groups (hypoparathyroid and control) had similarly low vitamin D levels [12]. This was also reported in another study dedicated to the correlation between preoperative vitamin D levels and postoperative hypocalcemia, with the results showing that decreased levels could not predict postoperative hypocalcemia and higher levels could not prevent it [13]. Severe deficiency showed a trend for transient hypoparathyroidism but not for permanent cases [13]. Furthermore, two studies from Spain outlined the opposite effect, revealing that decreased levels of preoperative vitamin D resulted in a lower incidence of postsurgical hypoparathyroidism, and this may act as a preconditioning factor for parathyroid gland recovery after thyroid surgery [14,15]. Based on all these confusing results about vitamin D, we chose to perform a cohort study based on clinical observations about sunshine exposure and postoperative hypoparathyroidism, abandoning the complicated mechanism of vitamin D metabolism altogether.

As concluded from our study, preoperative serum ionized calcium levels and postoperative parathyroid hormone levels are correlated with sunshine levels. A study by Ru et al. reported a correlation between preoperative calcium levels and temporary hypoparathyroidism and postoperative levels of parathyroid hormone, serving as a risk factor for permanent hypoparathyroidism [16]. The role of preoperative levels of calcium and pre- and postoperative parathyroid hormone levels in predicting hypoparathyroidism is already established [3,8,17,18]. A meta-analysis from 2024 reported that only preoperative levels of calcium constitute a risk factor for hypoparathyroidism, whereas preoperative parathyroid hormone levels could be a protective factor [19]. The role of sunshine exposure itself in this correlation has been implied by our study.

### Strengths and Limitations

The limitations of our study should also be pointed out. First of all, although we included 25-hydroxy- vitamin D levels in our study, the aim of our study was not to correlate vitamin D levels with the occurrence of postoperative hypoparathyroidism, as these levels can be affected by nutrition or preoperative supplementation. Moreover, we matched our patients at each center, including preoperative vitamin D supplementation if provided. The aim of this study was to investigate the influence of sunlight exposure alone on postoperative hypoparathyroidism. Furthermore, the limited number of participants in our study is also a limitation, and, therefore, larger studies should be conducted to thoroughly clarify this subject.

## 5. Conclusions

Sunshine levels and sunlight exposure can affect biochemical factors related to postoperative hypoparathyroidism after thyroid operations. Preoperative levels of serum ionized calcium and phosphorus and postoperative levels of parathyroid hormone and phosphorus seem to be correlated with different levels of sunshine throughout the year depending on weather conditions in different locations. The results are preliminary and hypothesis-generating due to the small cohort size. Further studies must be conducted to confirm and evaluate the potential contributors to these results.

## Figures and Tables

**Table 1:** Demographic and clinical data for each group, with no statistically significant differences between groups (m: male, f: female, TT: total thyroidectomy, TT + CC: total thyroidectomy with central compartment lymph node dissection, and TT + CC + LC: total thyroidectomy with central and lateral compartment lymph node dissection).

	Group T	Group R	*p* Value (<0.05)
Age (y/o)	48.17 ± 13.08	47.70 ± 13.04	0.99
Gender (M:F)	18:42	17:43	0.84
Diagnosis (benign/ malignant)	30:30	32:28	0.71
Type of operation (TT:TT + CC:TT + CC + LC)	39:17:4	39:15:6	0.76
25(OH) Vitamin D (ng/mL)	23.44 ± 9.87	28.64 ± 8.32	0.87
Vitamin D status			
Deficiency (<20 ng/mL)	27/60 (45%)	18/60 (30%)	
Insufficiency (20–30 ng/mL)	28/60 (46.6%)	27/60 (45%)	
Optimal (>30 ng/mL)	5/60 (8.3%)	15/60 (25%)	
Oral vitamin D supplementation	5/60	1/60	0.2

## Data Availability

Data will be available after a reasonable request is made.
